# Sensorimotor impairment and haptic support in microgravity

**DOI:** 10.1007/s00221-020-06024-1

**Published:** 2021-01-19

**Authors:** Bernhard Weber, Cornelia Riecke, Freek Stulp

**Affiliations:** German Aerospace Center, Institute of Robotics and Mechatronics, Oberpfaffenhofen, Germany

**Keywords:** Microgravity, Force feedback, Haptic interfaces, Sensorimotor performance

## Abstract

Future space missions envisage human operators teleoperating robotic systems from orbital spacecraft. A potential risk for such missions is the observation that sensorimotor performance deteriorates during spaceflight. This article describes an experiment on sensorimotor performance in two-dimensional manual tracking during different stages of a space mission. We investigated whether there are optimal haptic settings of the human-machine interface for microgravity conditions. Two empirical studies using the same task paradigm with a force feedback joystick with different haptic settings (no haptics, four spring stiffnesses, two motion dampings, three masses) are presented in this paper. (1) A terrestrial control study ($N=20$ subjects) with five experimental sessions to explore potential learning effects and interactions with haptic settings. (2) A space experiment ($N=3$ cosmonauts) with a pre-mission, three mission sessions on board the ISS (2, 4, and 6 weeks in space), and a post-mission session. Results provide evidence that distorted proprioception significantly affects motion smoothness in the early phase of adaptation to microgravity, while the magnitude of this effect was moderated by cosmonauts’ sensorimotor capabilities. Moreover, this sensorimotor impairment can be compensated by providing subtle haptic cues. Specifically, low damping improved tracking smoothness for both motion directions (sagittal and transverse motion plane) and low stiffness improved performance in the transverse motion plane.

## Introduction

Space agencies around the world are planning planetary exploration missions with robotic systems that are tele-operated by humans. To avoid exorbitant telecommunication delays during Moon or Mars missions, these robots will not be controlled from Earth but from orbital spacecraft (Anderson et al. 2020). Here, the question arises whether and to which degree human operators will be capable of performing such missions with the highest accuracy when being exposed to microgravity conditions. Although astronauts are intensively trained to cope with these conditions, there is ample empirical evidence documenting a significant loss of sensorimotor performance in microgravity (e.g., Lackner and DiZio 2000; Manzey 2017). Aiming motions, e.g., are slowed down, decreased accuracy has been reported for smooth tracking motions (Kanas and Manzey 2008) as well as for force production tasks (Hermsdörfer et al. 1999, 2000), and these sensorimotor impairments are most evident in the initial phase of exposition to microgravity. Space missions pose numerous challenges to the human operator and not surprisingly there have been diverse explanatory approaches for the deterioration of sensorimotor performance. Two main explanatory approaches have been discussed for sensorimotor impairments during space flight: microgravity-related disturbances of the sensorimotor systems [e.g. distorted proprioception (Lackner and DiZio 2000)] and mission-related workload and associated attentional deficits (Manzey et al. 2000).

When disentangling the mechanisms underlying impaired sensorimotor performance in microgravity, moderating variables like the temporal pattern of emergence (Manzey et al. 2000) or individual vulnerability to microgravity effects (Bock 1998; Kornilova 1997) have to be considered.

## Underlying mechanisms and moderators of impaired sensorimotor performance in microgravity

### Underlying mechanisms

Sensorimotor performance decrements during space-flight have been linked to the lacking gravitational force and related physiological changes. Afferent sensory signals from proprioceptors in muscles and joints as well as the vestibular system are known to be distorted in microgravity (e.g. Kanas and Manzey 2008; Lackner and DiZio 1992). Particularly, muscle spindle sensitivity which is crucial for proprioception is altered by the weightlessness of the body and limbs (Lackner and DiZio 2000), because muscles remain passive and hence spindles go slack (Proske 2019). Moreover, it has been discussed that the unloading of otolith organs in microgravity also affects the vestibulo-spinal modulation of the muscle spindle sensitivity (Lackner and DiZio 1992).

The sensorimotor system has to adapt to this lack of valid sensory input (Bock 1998; White et al. 2020; Clément et al. 2020) in the first weeks of spaceflight (Kanas and Manzey 2008). Accordingly, Manzey and his colleagues (Manzey et al. 2000, 1993, 1998) documented that performance decrements during a manual tracking task in the initial phase of exposure to microgravity and attributed these decrements to the incomplete adaptation to microgravity.

However, impairments of sensorimotor performance have also been reported to sometimes reemerge in later mission phases when sensorimotor adaptation is usually completed (Kanas and Manzey 2008). Researchers argued that decreased attentional resources due to general mission-related stressors are the underlying mechanism in this case (Manzey et al. 2000, 1993; Bock et al. 2003; Fowler et al. 2008). While the discussed explanatory approaches make divergent predictions regarding the temporal pattern of emergence, both have in common that individual resources are crucial for maintaining sensorimotor performance despite distorted proprioception or increased general workload. Consistently, Bock and his colleagues (Bock 1998; Bock et al. 2003, 2010) emphasized that maintaining performance in microgravity is a resource-demanding process and may vary with the computational demands of a specific task [also see White et al. (2020)] and the individual amount of resources (pre-mission training levels and resource availability during the mission).

### Haptic support and sensorimotor performance

Besides temporal and individual moderators, the current work focusses on the potential moderating influence of haptic support at the human-machine interface. In terrestrial applications, sensorimotor performance is usually supported by appropriate haptic settings. Joysticks, e.g., usually have a mechanical spring implemented to facilitate re-centering and to stabilize joystick deflections. Moreover, viscous motion damping and the joystick handle’s mass also have an impact on motion accuracy. Indeed, studies documented beneficial effects of moderate stiffness on tracking performance (Jones and Hunter, 1990; Weber and Rothkirch 2014). Damping and mass smoothen motions and prevent unintended jerk or tremor-like oscillations during tracking (Jones and Hunter 1993; Lange 2014; Bahrick et al. 1955). Besides supporting terrestrial performance, haptic support is a promising candidate for maintaining performance during spaceflight. Even subtle haptic cues help improving body stabilization (Riley et al. 1999) particularly when the vestibular function is impaired (Lackner and DiZio 2018). Indeed, the authors (Weber et al. 2020) could document that the positive effect of motion damping is also evident when performing tracking tasks in simulated weightlessness (induced by shallow water immersion). More interestingly, the same study also provided evidence that the sensorimotor impairment in weightlessness was mainly due to distorted proprioception and can be mitigated by applying low spring stiffness. Seemingly, very subtle haptic cues have the potential to improve proprioception in simulated weightlessness. Bringoux et al. (2012) also documented that elastic bands producing a torque on the shoulder during arm-reaching movements, allowed subjects to perform under conditions of microgravity (parabolic flight) as accurately as under normal gravity conditions.

The present study is based on the findings of the recently conducted underwater study (Weber et al. 2020) and investigates manual tracking performed at different phases of a space mission using a joystick with similar haptic settings (no haptics $=$ isotonic, four stiffnesses, two dampings, three virtual masses). Based on the considerations and evidences above, we formulated the following hypotheses:

***H1****: Microgravity-induced sensorimotor impairments are most evident in the initial phase of adaptation.*

No formal hypothesis is formulated regarding potential effects due to attentional deficits, since the occurrence and individual impact of mission-related stressors cannot be predicted.

The influence of individual resources on sensorimotor impairment and adaptation to microgravity will be explored in the light of cosmonauts’ sensorimotor performance capabilities.

Moreover, we assume that the negative effect of microgravity should be moderated by haptic settings:

***H2****: Haptic settings moderate the magnitude of sensorimotor impairments in microgravity. Specifically, damping and low stiffnesses should improve tracking performance in microgravity conditions.*

Again, individual effects are expected, and the degree to which these haptic settings are helpful might vary across subjects. In addition to stiffness and damping, the authors explored in previous studies, different (virtual) masses were implemented at the joystick, i.e. inertial moments were varied, too. However, it remains unclear whether the same beneficial effects reported for damping will also be evident for these masses in microgravity conditions. Thus, no formal assumption will be formulated.

Two experiments were conducted to test the above hypotheses: (1) a terrestrial study ($=$ control study) to investigate time effects (e.g., learning) on sensorimotor performance during manual tracking and potential interactions with the explored haptic settings, and (2) a space experiment with three cosmonauts ($=$ main study) performing manual tracking under terrestrial (pre- and post-mission sessions) and microgravity conditions on board the ISS (after 2, 4, and 6 weeks of exposure).

The main contributions of this paper are (I) a better understanding of the when and why of sensorimotor performance impairments occurring in microgravity conditions and (II) practical advice which haptic settings of a human-machine interface are the most promising candidates for future space missions requiring highest manual precision in altered gravity conditions.

## Method

### Apparatus

**Fig. 1:**
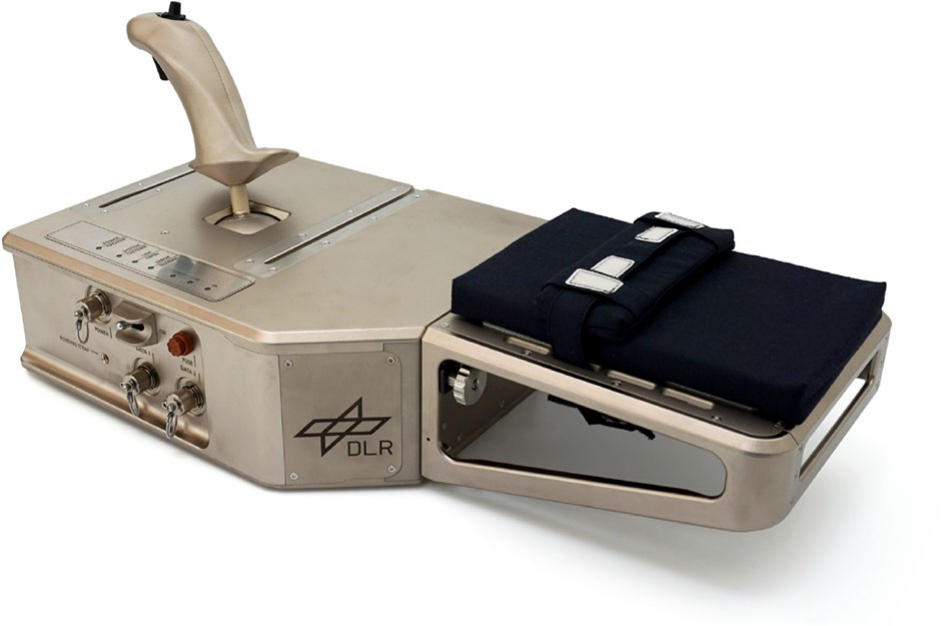
Force feedback joystick

A force feedback joystick developed at the German Aerospace Center (Riecke et al. 2016; workspace of $\pm 20^{\circ }$ for each axis; maximum force of 15*N*) was used as an input device (see Fig. 1). For the present experiments, a motion at the joystick was scaled up by a factor of 1:2, i.e. the required experimental workspace was fully covered with joystick deflections of $\pm 10^{\circ }$ for both axes. The joystick had an armrest with a padded elbow strap. This strap had to be attached to the elbow, ensuring that arm orientation and position was comparable across subjects and measurements. Still, the forearm could be moved without any restrictions within the required range of motion. The joystick was connected to a Lenovo T61P-6457 notebook. The experimental GUI was displayed on the 15.4” TFT screen of the notebook.

### Experimental task

**Fig. 2:**
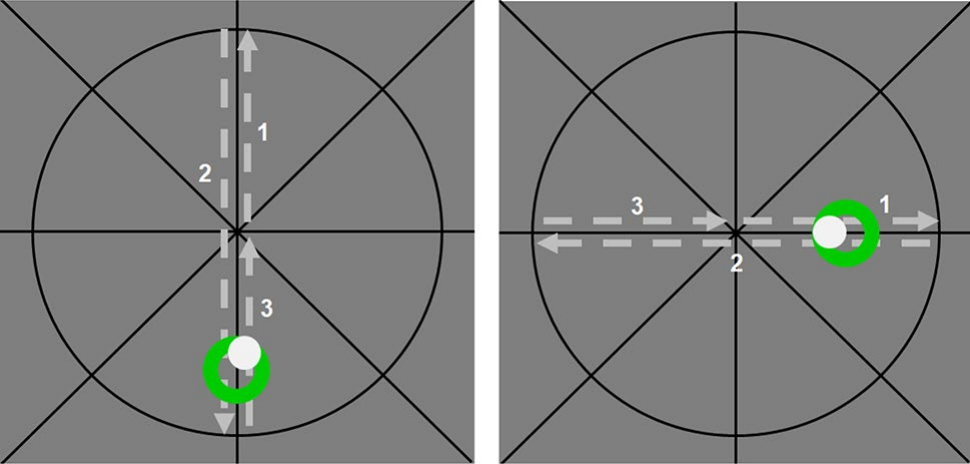
Experimental GUI with the two vertical and horizontal tracking tasks (target motion sequence in dashed lines). The light gray cursor had to be matched with the green target ring. Tracking error (RMSE of cursor-target deviations) and path lengths were calculated as performance measures

Subjects performed zero-order (i.e. joystick deflection equals cursor position), stable tracking tasks. The target moved with a constant speed of 13 mm/s along a vertical or a horizonal axis in the experimental GUI. Subjects controlled the cursor by moving the force feedback joystick with their right hand. The black circular cursor had to be moved to the starting point at the center of the crosshairs displayed in the GUI (see Fig. 2). Upon reaching the starting point, the cursor color turned green and a countdown appeared. The starting position had to be held for 2 sec, then the countdown disappeared, the cursor color turned gray and the green target ring started moving along the vertical or horizontal axis. The order of both motion directions was randomized. Participants were required to match the inner of the target ring as accurately as possible. This, of course, implies a minimization of cursor-target deviations as well as a smooth tracking of the constantly moving target. For both motion axes, the target ring moved from the starting point to the two intersections of axis and circle (corresponds to a joystick deflection of $\pm 8^{\circ }$) and returned to start (see Fig. 2). One complete tracking path length was 26 cm in the GUI and each task lasted 20sec. The upcoming tracking direction was always indicated by a quick target motion preview.

### Experimental design and procedure

In each of the five experimental sessions, ten experimental conditions with nine haptic conditions and an isotonic condition (no haptics) had to be completed (see Table 1). The haptic settings were the same we used in prior studies (Weber et al. 2020; Lange 2014). Since there were three subjects in the main study and three different haptic categories (stiffness, damping, virtual mass), a $3\times 3$ Latin Square design was utilized. Accordingly, three different category orders were defined (1: stiffness, damping, mass; 2: damping, mass, stiffness; 3: mass, stiffness, damping). The individual stiffness, damping and virtual mass conditions, however, were conducted in the fixed order (from low values to high values) and the isotonic condition was always the fifth condition. In the first session, subjects were informed about the experiment and procedure by the experimenter and signed an informed consent form. Before starting an experimental session, subjects read a detailed description of the experimental task on the GUI. In each of the ten experimental conditions, there was a training trial with a randomly chosen tracking subtask (horizontal or vertical), to familiarize with the respective haptic setting. Subsequently, the experimental vertical and horizontal tracking tasks were performed. Subjects rated the perceived workload after each experiment (“Please rate your overall workload during the last task”, adopted from Vidulich and Tsang (1987), scale ranging from 1-20). A standardized test measuring sensorimotor coordination (Bauer et al. 2002) (SMK module of the Vienna Test System, 15 min test version) was completed one week before (in the terrestrial control study with 20 subjects) or directly after having completed the first experimental session (in the main study with three cosmonauts). The SMK assesses the coordination of eye-hand or hand-hand by maneuvering a circular segment which randomly deviates for a center position. The three motion dimensions of the segment (*x*, *y* translations and tilt) are controlled by two joystick-like devices.

**Table 1:** Experimental conditions and haptic settings

Isotonic	No haptics
Spring stiffness 1	0.075 Nm/rad
Spring stiffness 2	0.225 Nm/rad
Spring stiffness 3	0.375 Nm/rad
Spring stiffness 4	0.525 Nm/rad
Motion damping 1	0.045 Nm s/rad
Motion damping 2	0.090 Nm s/rad
Virtual mass 1	0.0015 kg m$^2$
Virtual mass 2	0.0023 kg m$^2$
Virtual mass 3	0.0030 kg m$^2$

### Control study on time effects

#### Sample and setup

Twenty subjects (6 females, 14 males; $M = 36.4$ (11.0) years) voluntarily participated in the study, which was conducted at the German Aerospace Center in Oberpfaffenhofen. After having signed an informed consent document, subjects were seated at a desk and individually adjusted their seat height so that their right forearm rested comfortably on the joystick’s armrest. An adjustable laptop stand was used to have the same relative position and distance of the notebook from the subject like on board the ISS.

#### Experimental schedule

After having performed the SMK test one week before the main experiment, subjects completed the five experimental sessions following the experimental schedule of the main study. Accordingly, subjects started with their first experimental session (*T*1) and follow-up sessions were scheduled 105 (*T*2), 118 (*T*3), and 132 days (*T*4) later. The last session deviated from the schedule and was terminated 162 days (*T*5; instead of 279 days) later to avoid a high drop-out rate. Subjects were assigned to the three category orders in a way that similar SMK test scores were achieved for each experimental group.

### Main study

#### Sample

Three cosmonauts (42, 45, and 53 years) participated in the experiment. Two already had previous space mission experience (143 and 164 days on board the ISS).

**Fig. 3:**
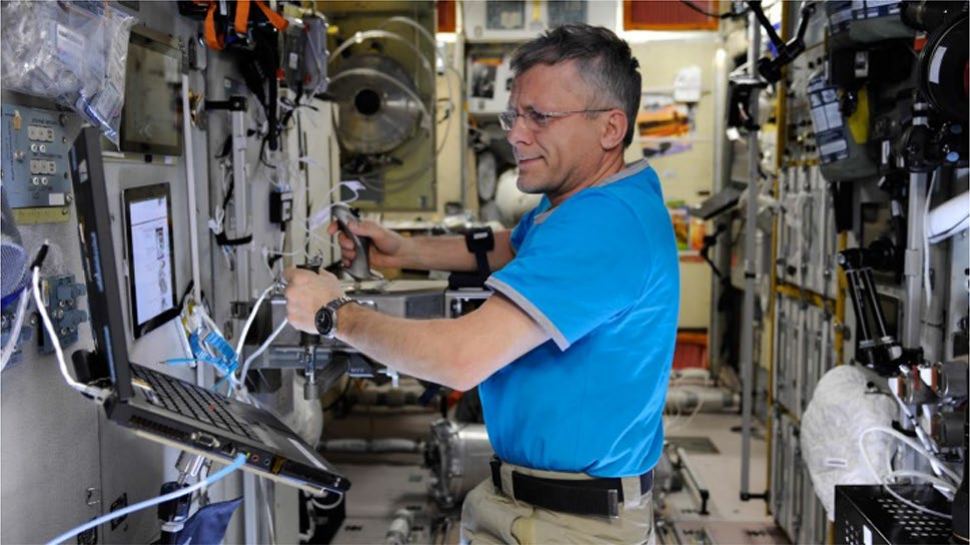
Experimental setup on board the Russian Zvezda module of the International Space Station. Cosmonaut performing system check

#### Experimental setup

The terrestrial pre- and post-mission sessions were conducted in the Gagarin Cosmonaut Training Center in Moscow. The setup was similar to the setup of the control study. For the mission sessions, the experimental setup with joystick and notebook was installed at a module wall of the Russian Zvezda service module of the ISS (see Fig. 3). Cosmonauts stabilized their body using a “foot” rail at the “bottom” and an additional grip for the left hand.

#### Experimental schedule

All of the three cosmonauts completed the same experiment during a pre-mission session (including the SMK test), 91 days before the mission launch (*T*1), three ISS sessions 14 (*T*2), 27 (*T*3) and 41 (*T*4) days after Soyuz docking and a post-mission session, 15 days after the half-year space mission (*T*5).

### Data analysis

For the SMK test, the time in ideal range was calculated as a main measure of sensorimotor coordination ability. This is the proportion of time (in %) the subject was able to hold the controlled segment in the target position with a threshold range of $\pm 25$ pixels for translational and ± 25$^{\circ }$ for tilting movements. The raw values are interpreted with reference to a comparative sample ($N = 239$ adults) in form of a percentile rank (PR).

Data of both studies were recorded with a sampling rate of 100 Hz. The square root of the mean squared distances between cursor and target (RMSE) was calculated as tracking error. Additionally, the overall length of the cursor trajectory was measured as an indicator for small-range corrective motions (e.g. oscillating movements) and thus motion smoothness.

For the control study, both performance metrics were analyzed performing a repeated measures Session * Haptic Setting * Tracking Direction ANOVA (rmANOVA). Workload ratings were analyzed performing a rmANOVA with Session as within factor. In case of non-sphericity, Greenhouse–Geisser corrections (GG.) were applied. Post-hoc comparisons were performed using the Bonferroni correction.

For the main study, Quade tests (Quade 1979) were performed on all metrics as a non-parametric alternative to rmANOVA. Repeated measures are ranked within each case and the deviation from the expected rank is weighted by the cases’ ranking of measure range. Post-hoc comparisons were calculated using the procedure suggested by Conover (1980). The $\alpha$ levels of post-hoc comparisons were adjusted using the Benjamini–Hochberg procedure (with a FDR of 0.1; Benjamini and Hochberg (1995)). The raw *p*-values are reported and it is indicated if significance was not reached after $\alpha$ adjustment.

For both performance metrics, **H1** was tested with Quade tests on Session and subsequent post-hoc tests and **H2** by analyzing Haptic Setting effects in each experimental session and subsequent post-hoc comparisons. Finally, workload ratings were analyzed by a Quade test on Session and corresponding post-hoc tests.

## Results

### Control study on time effects

#### Sensorimotor coordination

Subjects reached an average PR of 74.7 (18.0).

**Fig. 4:**
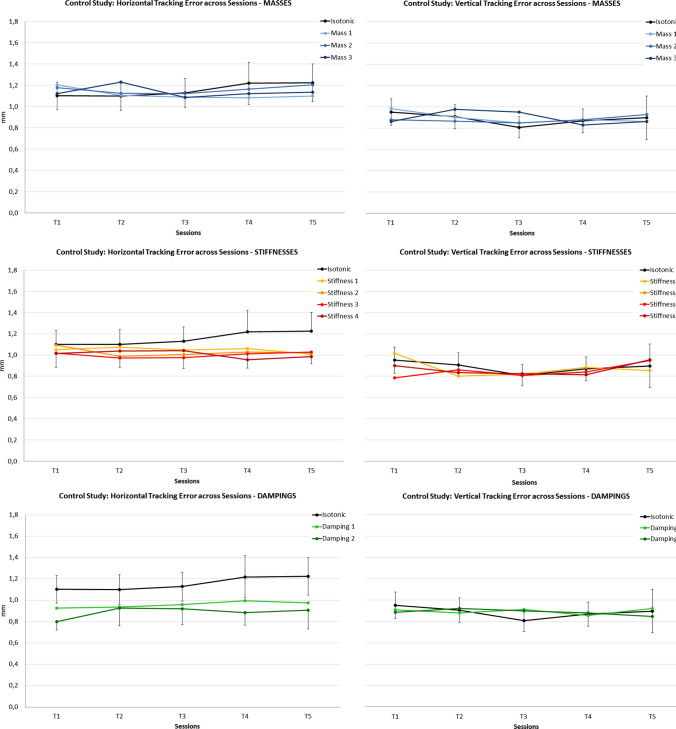
Tracking errors in the control study: Overview of the haptic setting effects compared to the isotonic baseline condition (black line) in the five experimental sessions averaged across subjects ($T1 =$ pre-mission, *T*2–*T*4 $=$ mission, $T5 =$ post-mission session. Left column: horizontal tracking; Right column: vertical tracking). 95% CI intervals are displayed for the isotonic condition and partially for selected conditions to avoid visual cluttering

#### Tracking error

RmANOVA on RMSE revealed no significant overall Session effect. However, Tracking Direction (*F* (1, 19) $=$ 127.5; *p* < 0.001) and Haptic Setting (*F* (4.02, 76.4) $=$ 5.0; *p* $=$ 0.001; GG.) main effects were evident. Furthermore, a significant Tracking Direction * Haptic Setting interaction effect occurred (*F* (9, 19) = 9.8; *p* < 0.001). RMSE was significantly higher for the horizontal compared to the vertical tracking direction and significant effects of the haptic setting were only evident for the horizontal tracking task (see Fig. 4). Here, post-hoc contrasts with Bonferroni correction revealed significantly lower tracking errors for Stiffness 3 and 4 (*p* < 0.01; *p* < 0.05) as well as for both Damping 1 and 2 (*p* $=$ 0.001; *p* < 0.001) compared to the isotonic baseline condition.

**Fig. 5:**
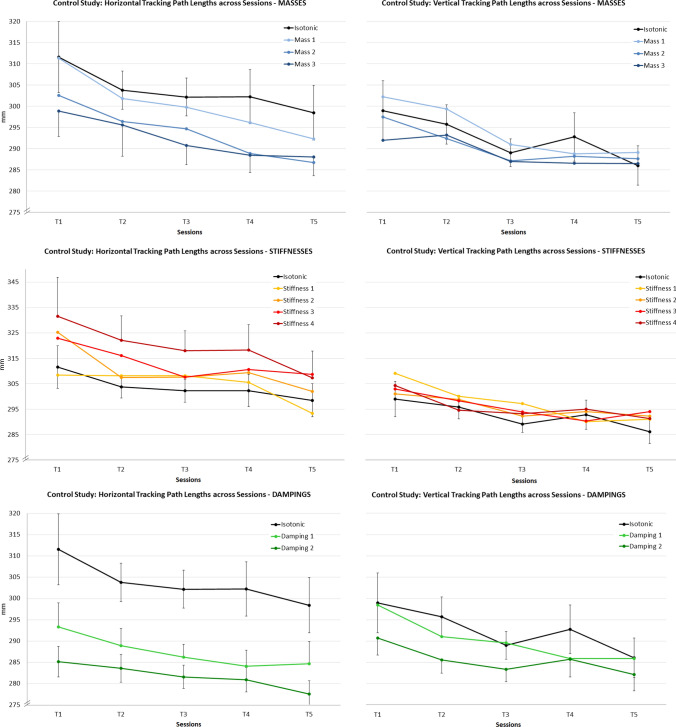
Path lenghts in the control study: overview of the haptic setting effects compared to the isotonic baseline condition (black line) in the five experimental sessions averaged across subjects ($T1 =$ pre-mission, *T*2–*T*4 $=$ mission, $T5 =$ post-mission session. Left column: horizontal tracking; Right column: vertical tracking). 95% CI intervals are displayed for the isotonic condition and partially for selected conditions to avoid visual cluttering

#### Path lengths

The same rmANOVA on path length yielded significant main effects for Session (*F* (2.5, 48.1) = 14.8; *p* < 0.001; GG.), Haptic Setting (*F* (2.1, 39.9) = 22.7; *p* < 0.001; GG.) and Tracking Direction(*F* (1, 19) = 31.6; *p* < 0.001). Learning effects were evident for both motion directions: Horizontal path length averaged across all conditions decreased successively [*T*1 = 309.1; *T*2 = 302.4; *T*3 = 300.1; *T*4 = 298.5 mm (*p*$_{({T1{-}T4})}<$ 0.05); *T*5 = 293.9 mm (*p*$_{({T1-T5})}<$ 0.001)]. For the vertical tracking, a similar trend was found [*T*1 = 299.7; *T*2 = 294.9; *T*3 = 290.3 mm (*p*$_{{(T1{-}T3)}}<$ 0.01); *T*4 = 289.7 mm (*p*$_{{(T1{-}T4)}}<$ 0.001); *T*5 = 288.6 mm (*p*$_{{(T1{-}T5)}}<$ 0.01)]. As for RMSE, a significant Tracking Direction * Haptic Setting interaction effect occurred (*F* (4.4, 83.4) = 24.8; *p* < 0.001, GG.). Path lengths were longer for horizontal compared to vertical tracking and steadily decreased for both directions across sessions. Moreover, the haptic setting effects were more evident for horizontal compared to vertical tracking (Fig. 5). Post-hoc contrasts with the isotonic condition revealed significantly longer paths for stiffnesses 3 and 4 (*p* < 0.01; *p* < 0.01) and significantly shorter paths for damping 1 and 2 (*p* < 0.001; *p* < 0.001) as well as for virtual mass 2 and 3 (*p* < 0.05; *p* < 0.001) in the case of horizontal tracking. For vertical tracking, paths were significantly shorter in the damping 2 condition only (*p* < 0.01).

#### Overall workload

No significant Session effect was evident in rmANOVA on workload ratings (*T*1: 7.2; *T*2: 7.1; *T*3: 6.9; *T*4: 7.1; *T*5: 6.9; $F(2.4, 45.5) = 0.88$, ns., GG.).

#### Summary

The main findings of this control study are: (1) horizontal tracking performance is worse than vertical tracking, probably due to the worse stabilization when rotating the forearm in the transverse motion plane [see Weber et al. (2020)]. (2) Higher stiffnesses and damping significantly reduce tracking error for the horizontal tracking task. (3) However, path lengths were significantly increased with higher stiffnesses, i.e. the improvement of tracking error came at the cost of decreased motion smoothness during horizontal tracking. Providing higher virtual masses and dampings is beneficial to smoothen the horizontal tracking motions and even vertical tracking was improved by high damping. (4) Time effects were evident for path lengths, with a linear learning trend for both motion directions, i.e. subjects learn to better stabilize their movements.

### Main study

#### Sensorimotor coordination

The three cosmonauts achieved SMK scores of PR$_\text {Cosm. 1}$ = 41 (95%-CI: 31-54), PR$_\text {Cosm.2}$ = 57 (95%-CI: $46-69$), and PR$_\text {Cosm. 3}$ = 85 (95%-CI: $76-90$). While the former two values can be interpreted as being average, the latter one is clearly above average.

#### Tracking error

**Fig. 6:**
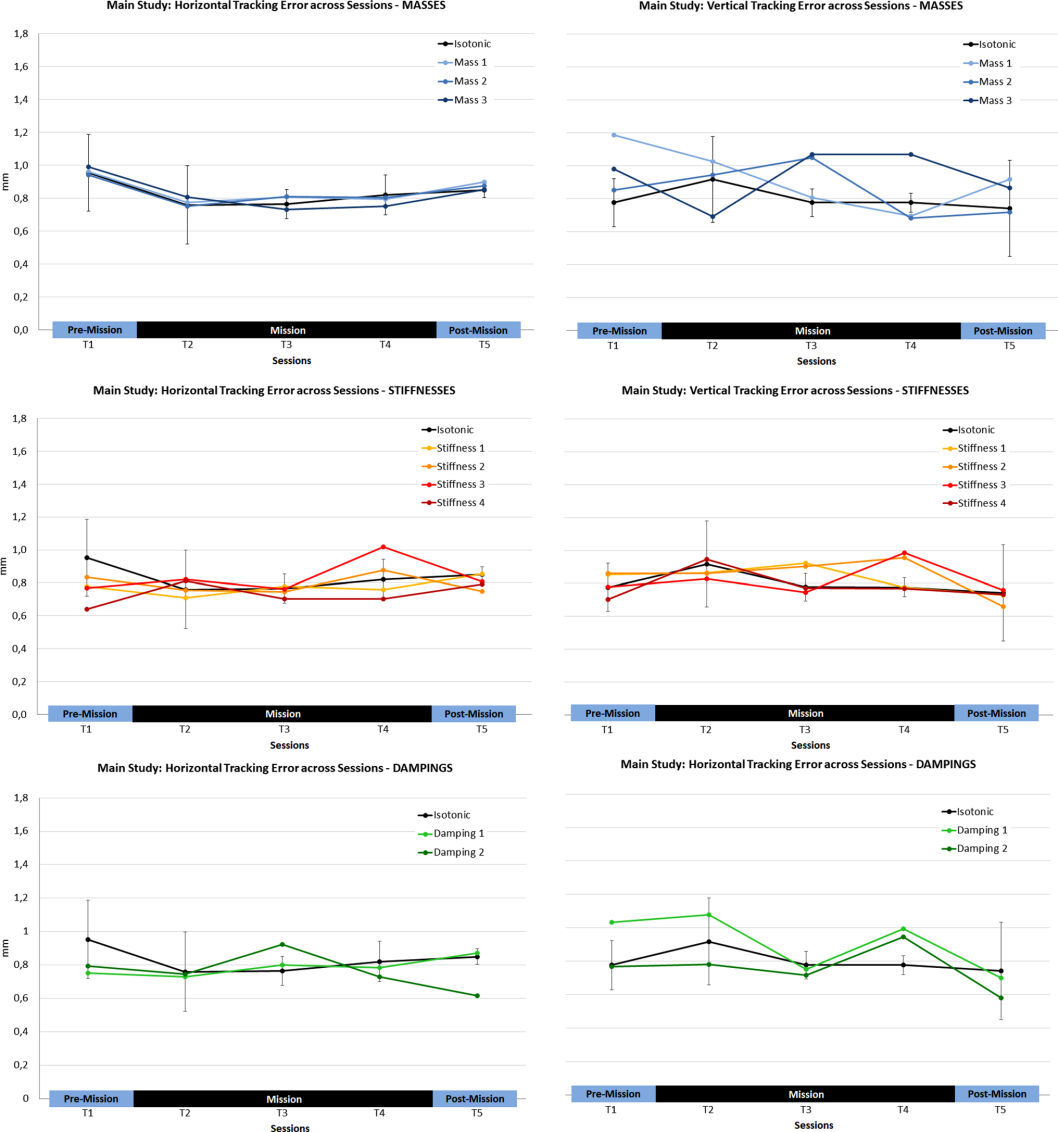
Tracking errors in the main study: overview of the haptic setting effects compared to the isotonic baseline condition (black line) in the five experimental sessions averaged across cosmonauts (*T*1 = pre-mission, *T*2–*T*4 = mission, *T*5 = post-mission session. Left column: horizontal tracking; Right column: vertical tracking. 95% CI intervals are displayed for the isotonic condition

Based on the findings of the control study, we did not expect time effects for the RMSE, the expected rank in the Quade test was simply the average rank (= 3). Here, no Session, Haptic Setting or interaction effects were evident. Moreover, no significant effect was found when comparing both Tracking Directions with the Quade procedure (see Fig. 6). Contrary to the findings of the control study, cosmonauts were able to stabilize horizontal tracking motions (*M* = 0.80 mm; SD = 0.06 mm) as well as vertical tracking motions (*M* = 0.85 mm; SD = 0.06 mm).

#### Path lengths

First, Session effects were tested. As we found a learning trend for path lengths across sessions in the control study, the expected ranking for the five consecutive sessions in the Quade test was “5, 4, 3, 2, 1”.

**Fig. 7:**
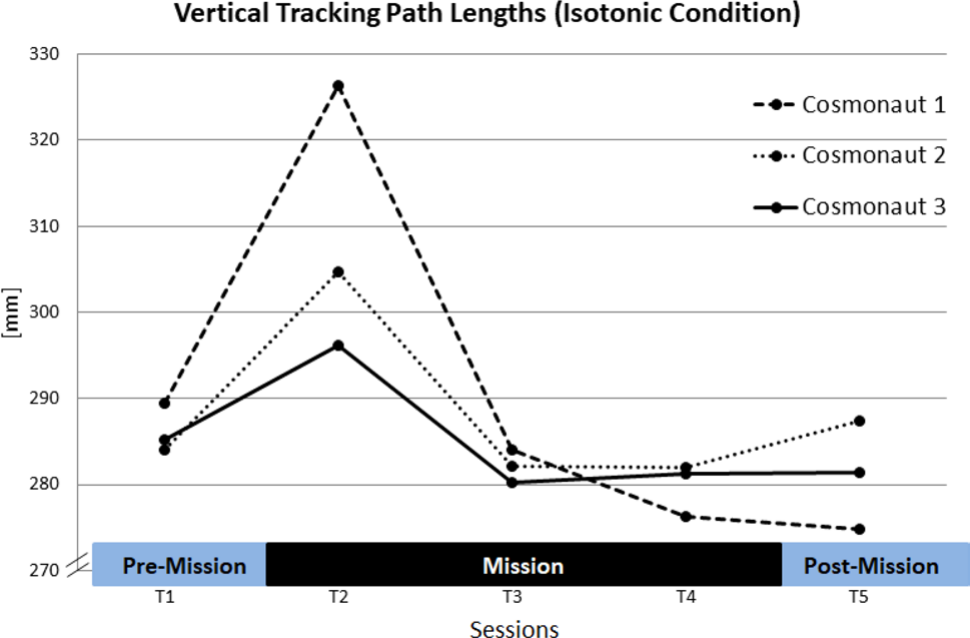
Vertical tracking path lengths for individual cosmonauts in the isotonic conditions

While no overall Session effect was found, analyzing the results for the isotonic baseline condition solely revealed a Session effect for the vertical tracking subtask: Here, a significant overall effect occurred (*F* (4, 8) = 4.0; *p* < 0.05), with significantly increased path lengths in session T2 and significantly decreased path lenghts in *T*5 compared to session *T*1 (*T*1–*T*2: *t*(8) = 2.92; *p* < 0.05; *T*1–*T*5: *t*(8) = 3.34; *p* $=$ 0.01). Please note that vertical tracking path lengths of all cosmonauts were longest in the first session on board the ISS (T2) (see Fig. 7).

Next, the overall Haptic Setting effect was explored (with average ranks expected), and a significant effect was found for the horizontal tracking subtask (*F* (9,18) = 5.3; *p* $=$ 0.001). Based on the findings of the control study, we expected higher stiffnesses to have a negative and damping as well as higher masses to have a positive effect. Indeed, stiffnesses 3 and 4 again led to longer path lengths compared to the isotonic baseline condition during horizontal tracking (Stiffness 3: *t*(18) = 2.44, *p* < 0.05; Stiffness 4: *t*(18) = 3.03, *p* < 0.01; one-tailed testing [1tt]). Moreover, there was a non-significant trend for damping 1 (*t*(18) = 2.44, *p* < 0.10; 1tt), i.e. horizontal path lengths tended to be shorter compared to the isotonic condition.

**Fig. 8:**
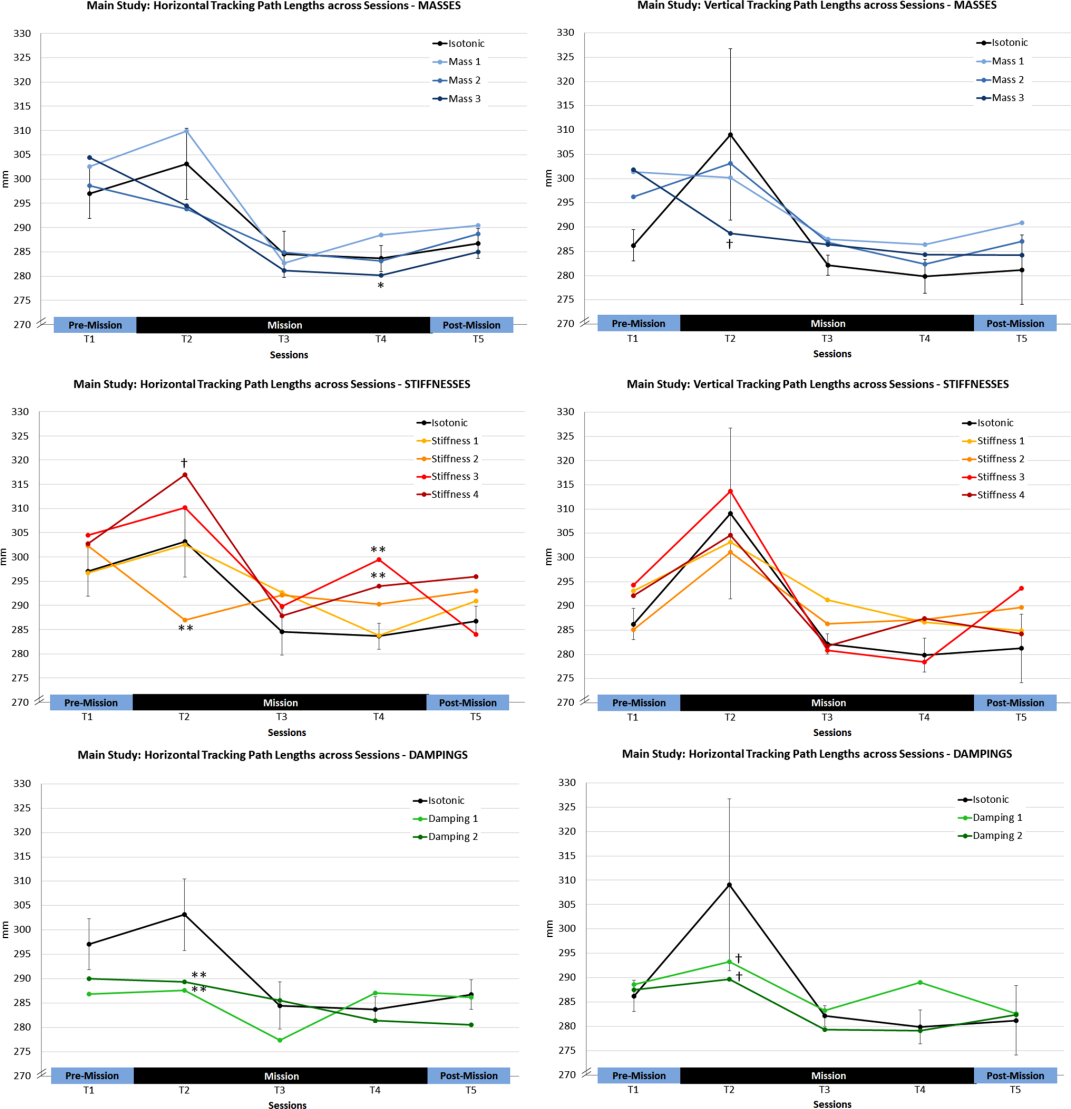
Path lengths in the main study: overview of the haptic setting effects compared to the isotonic baseline condition (black line) in the five experimental sessions averaged across cosmonauts (*T*1 = pre-mission, *T*2–*T*4 = mission, *T*5 = post-mission session. Left column: horizontal tracking; Right column: vertical tracking. 95% CI intervals are displayed for the isotonic condition and partially for selected conditions to avoid visual cluttering. $${}^\dagger$$*p* < 0.10; $$*_p$$ < 0.05; $$**_p$$ < 0.01)

In a final step, the potential interaction of Session and Haptic Setting was analyzed. For each of the five sessions, the path lengths of the different haptic setting were compared with the isotonic baseline condition (see Fig. 8 for an overview of results).

For the horizontal tracking task, overall effects were evident for sessions *T*2 (*F* (9, 18) = 6.1; *p* < 0.001) and *T*4 (*F* (9, 18) = 5.8; *p* < 0.001). When comparing the isotonic condition with the haptic settings in session *T*2, significantly shorter path lengths were found for stiffness 2 (*t* (18) = 3.0; *p* < 0.01) and non-significant tendency for longer path lengths in the stiffness 4 condition (*t* (18) = 1.8; *p* < 0.05, 1tt; ns. after $\alpha$ correction; see Fig. 8). Moreover, both dampings had the expected positive effect (both *t*s (18) = 2.6; *p* < 0.01, 1tt). In session T4, longer path lengths were evident for stiffness 3 (*t* (18) = 2.9; *p* < 0.01, 1tt) and 4 (*t* (18) = 2.7; *p* $=$ 0.01, 1tt) compared to the isotonic baseline. Additionally, a positive effect of mass 3 was found in this session (*t* (18) = 2.1; *p* < 0.05, 1tt).

Regarding the vertical tracking task, a significant effect of haptic settings was found for session *T*2 (*F* (9, 18) = 2.7; *p* < 0.05). Comparing dampings with the isotonic reference revealed a non-significant trend for shorter path lengths (*t*$_{\text {Isot-Damp1}}$(4) = 1.8, *p* < 0.05; 1tt; *t*$_{\text {Isot-Damp2}}$(4) = 2.4, *p* < 0.05, 1tt; both ns. after $\alpha$ correction), see Fig. 8. Finally, a similar tendency was found when comparing the isotonic and mass 3 conditions (*t*(18) = 2.0; *p* < 0.05; 1tt; *ns.* after $\alpha$ correction).

Finally, a Quade test comparing Tracking Directions revealed similar path lengths for horizontal (*M* = 291.9 mm; SD = 3.7 mm) and vertical tracking (*M* = 289.5 mm; SD = 1.2 mm), also see Fig. 8.

#### Overall workload

A significant Session effect was found when analyzing cosmonauts’ workload ratings (*F* (4,8) = 7.4; *p* < 0.01); post-hoc tests indicated that perceived workload increased in *T*2 (*T*1–*T*2: *t* (8) = 4.9; *p* < 0.01), *T*3 (*T*1–*T*3: *t* (8) = 3.4; *p* < 0.01) and *T*5 (T1-T5: *t* (8) = 3.2; *p* < 0.05) compared to *T*1 (*T*1: 3.8, *T*2: 6.1, *T*3: 5.2, *T*4: 4.4, *T*5: 5.1).

## Discussion

In the present work, impairments of sensorimotor performance in different stages of a space mission were investigated using a manual tracking task paradigm. Different haptic settings (stiffness, damping, virtual mass) of the human-machine interface were compared with conditions without haptic support (isotonic baseline condition). Prior to the study conducted on board the ISS, a terrestrial control study was conducted to identify potential time effects of tracking performance and interactions with the haptic settings.

### Findings of the control study

The findings of the control study investigating tracking performance across five experimental sessions with twenty subjects, showed that tracking errors were larger when performing tracking motions in the transverse plane of motion (horizontal tracking in the GUI) and are significantly improved by applying higher stiffnesses (0.375–0.525 Nm/rad) as well as motion damping (0.045–0.090 Nm s/rad). However, these stiffnesses also lead to increased pathlengths, because it is more difficult to move against the spring-like forces in a smooth manner. Subjects were better able to perform smooth transverse tracking motions with higher masses (0.0023–0.003 kg m$^2$) and both dampings. Higher damping (0.090 Nm s/rad) also improved motion smoothness during tracking in the sagittal motion plane (vertical tracking in the GUI). Interestingly, this pattern was stable across the five experimental sessions and no interactions between haptic settings and sessions were evident. Finally, we found no time effects for the tracking error, but for motion smoothness. Seemingly, subjects learn to achieve positional tracking precision quickly, while stabilizing the flow of movement is incrementally improved across time with the best results in the final experimental session.

### Sensorimotor impairment in the early phase of adaptation to microgravity (H1)

In the main study, the impact of microgravity on sensorimotor performance was investigated with data from three cosmonauts. Specifically, we explored whether there is evidence for impaired performance in the initial phase of exposure to microgravity (**H1**). Interestingly, no effect was manifest for tracking error. Cosmonauts reached a high-performance level for both motion directions in the first pre-mission session and maintained this level in all subsequent phases of their mission. While the tracking error reflects the average deviations from the desired target position, smoothness reflects the regularity of tracking. Without haptic support (isotonic baseline condition), a significant deviation from the expected learning trend was evident for tracking path lenghts in the sagittal plane (vertical tracking): here, all cosmonauts showed their worst performance during their first session in microgravity (mission day 14). Accordingly, cosmonauts’ workload ratings indicate that performing the tracking task during the initial adaptation to microgravity was significantly more demanding compared to normogravity conditions.

Further, it was expected that individual resources should have a moderating impact on microgravity effects. Cosmonauts’ sensorimotor coordination capabilities were measured as a key resource for the experimental task. Intriguingly, the magnitude of performance losses (path lengths) in the isotonic baseline condition were directly related to the cosmonauts’ sensorimotor capabilities: The cosmonaut with the lowest capability value (PR = 41) showed the largest increment of path length in the first mission session compared to the pre-mission reference for both transverse (+ 8.4%) as well as sagittal tracking motions (+ 12.8%). The cosmonaut with the moderate rank (PR = 57) showed moderate increases (transverse: + 2.5%; sagittal: + 7.2%) and the cosmonaut with the highest rank showed a decrease for the transverse direction ($-4.5$%) and the lowest increase for the sagittal plane (+ 3.8%).

Altogether, **H1** is at least partially confirmed and we found clear evidence for sensorimotor impairment in the initial phase of adaptation to microgravity, which showed the expected impact of individual task-related capabilities.

The intriguing question is, whether the reported result pattern also allows conclusions about the underlying mechanisms. Until today, the complex interaction of sensory and central functions when adapting the sensorimotor system to microgravity is not completely understood. However, the present study provides at least some initial evidence that a proprioceptive distortion might be a plausible explanation for the obtained results, like prior research investigating sensorimotor impairments in microgravity (Bock et al. 1992; Fisk et al. 1993; Manzey et al. 2000) or simulated weightlessness (Dalecki et al. 2012).

1. There is a clear convergence of findings of the current study and the cited underwater study, where the same tracking experiment was conducted in simulated weightlessness induced by shallow water immersion (Weber et al. 2020). Re-analyzing the data of this study, revealed similar impairments of motion smoothness (path length) during water immersion which were mainly evident for the sagittal motion plane. In the current study, effect sizes (Hedges’ *g*) when comparing the isotonic condition of pre-mission and first mission session are *g* = 0.50 for the transverse motion plane and *g* = 1.63 for the sagittal motion plane. A very similar pattern emerged in the underwater study when comparing land and water conditions (transverse plane: *g* = 0.38; sagittal plane: *g* = 1.03). Since gravity is still present during water immersion, vestibular functions as well as gravity-related central representations should not be affected (Brown 1961). Distorted proprioception due to the changed muscle resting tone of the weightless limbs (Dalecki et al. 2012) is more likely to be a relevant mechanism underlying the findings of both studies.
2. This anisotropy of the result pattern is also consistent with research investigating sensorimotor performance of patients without any proprioception e.g. caused by peripheral neuropathy. While such patients are still able to perform single-joint movements without substantial error, more complex motions involving multiple joints and thus intersegmental coordination are strongly affected (Pagano and Turvey 1998; Sarlegna and Sainburg 2009; Sainburg et al. 1993; Ghez et al. 1990). Deafferented subjects, therefore, fail to produce smooth and motions e.g. involving shoulder and elbow joint motions (Messier et al. 2003). This is consistent with the findings of the current study, showing that motions involving only one joint (forearm rotation in transverse plane) was less affected than motions involving multiple joints (wrist, elbow and shoulder motion in sagittal plane).
3. Although the cosmonauts were well stabilized in the current experiment (“foot” rail, grip for left hand, armrest with strap for right elbow) the findings could still be a result of insufficient postural stability which is known to impair suprapostural fine motor performance (Chen and Stoffregen 2012). Indeed, prior research showed that e.g. aiming performance was affected by microgravity and this has been explained by insufficient body stabilization. As a consequence, the CNS employs an adjusted control strategy, i.e. motions are slowing down (Mechtcheriakov et al. 2002). However, these effects should be evident regardless of the mission phase (Clément et al. 2020; Berger et al. 1997), should mainly occur during rapid arm motion and should be stronger the higher the inertial load of the involved limbs is, since these motions are particularly difficult to compensate in weightlessness. In contrast, we found microgravity effects during very slow tracking motions and stronger effects for the sagittal motion plane, mainly involving wrist joint rotation and no significant forearm motion as it is required for the tracking motion in the transverse plane.

Apart from the underlying mechanism, the question arises, why the main effect was solely reflected in decreased smoothness and not in increased tracking error like in other studies, e.g. Manzey et al. (2000). This might be due to the lower task demands of the utilized tracking task paradigm. In prior studies on tracking performance in microgravity, an unstable, first-order tracking task was used (e.g. Manzey et al. 2000, 1993; Bock et al. 2003, 2010; Manzey et al. 1995), here a stable, zero-order tracking task was implemented, i.e. cursor motions were deterministic and cursor position was directly controlled by the joystick’s deflection (i.e. position control). The lower task demands together with the superior performance level of the cosmonauts might well explain the lack of significant tracking errors in microgravity. The comparably low task demands might also be the reason why there was no evidence for any attentional deficit effect, which might have affected performance during the mission. Seemingly, task complexity moderates the impact of microgravity on tracking error as emphasized by Bock et al. (2010).

### Mitigating the microgravity effect with haptic support (H2)

Next, we scrutinized the potential moderation of sensorimotor impairments in microgravity by haptic settings (spring stiffness, viscous damping, virtual mass) of the human-machine interface. Specifically, we assumed that motion damping as well as lower stiffness should attenuate the documented sensorimotor impairments in the early mission phase (**H2**). Results provided evidence that damping has the expected positive effect on motion smoothness. While this effect emerged in both motion directions, the positive effect was particularly evident for the transverse motion plane. Here, cosmonauts could maintain their terrestrial performance level in their first session in microgravity.

A similar positive effect of low spring stiffness (.225 Nm/rad) was found for the transverse motion axis in the initial mission session. Here, all cosmonauts showed improved motion smoothness compared to the isotonic baseline, while quite the opposite was true for the highest stiffness (0.525 Nm/rad). Please note that the overall pattern of haptic setting effects in the first mission session was similar for all of the three cosmonauts, although the general performance level was again contingent on their sensorimotor capabilities.

Summarizing, these findings corroborate hypothesis **H2** and damping effectively helps maintaining sensorimotor performance during early adaptation to microgravity. Damping haptically augments velocity and motion irregularities (like tremor) are physically prevented. Thus, significant motion irregularities are basically filtered out. However, what is really remarkable here, is the fact that even with very subtle damping (0.045 Nm s/rad) the sensorimotor impairment almost disappeared. Thus, it is conceivable that the mere availability of a permanent counterforce caused this effect because muscle tone is inevitable increased and thus muscle spindle sensitivity and thus proprioception should also improve (Proske 2019).

Virtual mass prevents high acceleration and the effect direction should be comparable with damping, since an abrupt motion impulse is impossible with a high mass. Yet, there was only a trend for higher masses to be effective during the first mission session (sagittal motion), and after several experimental sessions in the last mission session (transverse motion). The overall pattern of findings indicates that the advantage of virtual mass only emerges after several trials with this haptic category (the highest mass was always the last trial in this category) or after intensive experimental experience. The overall decreased motion smoothness for the low mass is also consistent with this notion. Furthermore, virtual mass only provides haptic information during velocity changes, and thus might be less helpful compared to damping when the desired velocity is once reached.

A more interesting question arises with regard to low stiffness. Why did it eliminate the sensorimotor deficit during transverse motion only? Reanalyzing the results from the study on manual tracking in simulated weightlessness (Weber et al. 2020), also revealed that the reported positive effect of low stiffness was exclusively due to the results from transverse tracking. In general, transverse tracking motions are likely more demanding when performing the motion reversals at the turning points. Compared to the sagittal tracking motion, mainly requiring wrist joint rotation [radial and ulnar deviation, e.g. Berger and Garcia-Elias (1991)] and also elbow and shoulder joint motions (flexion and extension), transverse tracking is almost exclusively performed by forearm rotations with the elbow as pivot (pronation and supination, e.g. Kapandji (2001)). So stabilizing joystick deflections in this motion plane is more demanding in the current task paradigm and thus accurate information about limb position is particularly important. Stiffness is a haptic augmentation of deflection and seems to be the appropriate support in this case. When increasing the resistive forces, however, the effect reverses and performance degrades again. Obviously, a fine equilibration of haptic cues is necessary for microgravity conditions. Higher resistive forces, e.g., which are beneficial in normogravity conditions often turn out to be detrimental in microgravity or simulated weightlessness conditions (Weber et al. 2020, 2019). What is surprising here, is the disappearance and re-appearance of the negative effect of high stiffness on transverse tracking smoothness in the two subsequent mission sessions (27th and 41st mission day). It seems less plausible here that general workload triggered this effect, since cosmonauts’ performance in the isotonic and other haptic setting conditions with higher resistive forces (high mass, damping) were not affected. Additionally, no indication of generally increased workload in the final mission session was found. It is conceivable here that stabilizing tracking motions against the higher resistive forces is generally more difficult (cf. Weber et al. (2019), 45 days in space) or less convenient in microgravity conditions and thus cosmonauts focused more on tracking error optimization at the cost of higher motion irregularity.

### Limitations

As most studies investigating microgravity effects during spaceflight, the current study’s generalizability is substantially restricted due to the small sample size inherent in working with subjects on the ISS.

However, the convergence of findings from the current and the prior underwater study (Weber et al. 2020) provides some evidence that there seems to be a general potential to utilize haptic settings to improve sensorimotor performance in weightlessness. Another major drawback of the longitudinal design of the study are potential time effects. The control study served as a baseline to estimate the general learning curves, however, it is clear that transferring these results to a sample of cosmonauts can be questioned. Particularly the expected performance rank for the final session (*T*5) might be invalid, since the time schedule of the space study was not exactly replicated for this session in the control study.

Moreover, it was surprising that cosmonauts’ sensorimotor capability values as measured with the SMK module were comparably low for two of the three cosmonauts and the actual tracking performance level very high. First, this test had to be completed after the first experimental session which certainly caused fatigue. Secondly, cosmonauts are intensively trained to control the TORU system for manual docking of the Soyuz capsule, which also includes two joystick-like devices for the control of six DoF. The mapping of rotational and translational DoF was completely different in the SMK test system, which was particularly challenging for the cosmonauts.

## Conclusion

The present study provides further evidence that sensorimotor performance during a manual tracking task requiring very precise and continuous motions changes under conditions of microgravity. Although cosmonauts seemingly are able to maintain the positional error on a level comparable with terrestrial performance, motion smoothness is substantially deteriorated in the early phase of adaptation to microgravity. Individual resources which are crucial for this adaptative process do play a significant role and it was found that sensorimotor capabilities do have a moderating impact here. For future missions, a rapid adaptation might be particularly important, e.g. in scenarios where human operators are exposed to non-nominal gravity environments on Moon or Mars and have to perform amidst the ongoing adaptation to the altered conditions. Subtle haptic support in form of low stiffness and low damping provided at the human-machine interface seems to be the most promising candidates to maintain performance even under such adverse conditions.

## Data Availability

The data that support the findings of this study are available from the corresponding author upon reasonable request.
